# Einstellung von Patienten zu möglicher Telemedizin in der Ophthalmologie: Erhebung mittels Fragebogen bei Glaukompatienten

**DOI:** 10.1007/s00347-021-01501-6

**Published:** 2021-09-20

**Authors:** Sarah B. Zwingelberg, Karl Mercieca, Eva Elksne, Stephanie Scheffler, Verena Prokosch

**Affiliations:** 1 Zentrum für Augenheilkunde, Medizinische Fakultät und Universität zu Köln, Uniklinik Köln, Kerpener Str. 62, 50937 Köln, Deutschland; 2 Universitäts-Augenklinik Bonn, Ernst-Abbe-Str. 2, 53127 Bonn, Deutschland; 3 Department of Ophthalmology, Riga Stradins University, Pauls Stradiņš Clinical University Hospital 13, Pilsoņuiela, Riga, Lettland

**Keywords:** Telemedizin, Ophthalmologie, Glaukom, Artificial intelligence, SARS-CoV-2, Telemedicine, Ophthalmology, Glaucoma, Artificial intelligence, SARS-CoV‑2

## Abstract

**Hintergrund:**

Die COVID-19-Pandemie im Jahr 2020 und 2021 schränkt die Versorgung augenärztlicher Patienten vielfach ein. Teleophthalmologische Leistungen wie Videokonsultation oder medizinische Telefonberatungen könnten den Mangel an notwendigen Kontrollen bei chronischen Erkrankungen, zumindest teilweise, kompensieren. Teleophthalmologische Angebote sind jedoch in Deutschland aktuell noch deutlich unterrepräsentiert.

**Ziel der Arbeit:**

Um die Bereitschaft auf Patientenseite zur Telemedizin und virtuellen Klinik zu ermitteln, führten wir bei Patienten der Hochschulmedizin mit bekanntem Glaukom als chronische Erkrankung eine Umfrage mittels Fragebogen während der ersten Welle der COVID-19-Pandemie zum Thema Teleophthalmologie durch.

**Methoden:**

Es wurden 100 Patienten befragt. Der Fragenkatalog beinhaltete 22 Fragen mit Mehrfachwahl-Antwortmöglichkeiten. Als Einschlusskriterium galten das Vorhandensein eines Glaukoms als chronische Erkrankung, Alter über 18 Jahre sowie eine ausreichende sprachliche Verständigung zur Beantwortung der Fragen. Die Daten wurden anonymisiert erhoben, analysiert und ausgewertet.

**Ergebnisse:**

In der Patientenumfrage konnte aufgezeigt werden, dass eine hohe Bereitschaft zur Teleophthalmologie bei den Befragten im Bereich des Glaukoms als chronische Erkrankung vorhanden ist und diese in Anspruch genommen werden würde; 74,0 % der Befragten würden Telemedizin und virtuelle Kliniken akzeptieren; 54,0 % der Befragten ophthalmologischen Patienten gab an, dass ihr Arzt‑/Klinikbesuch aufgrund von SARS-CoV‑2 nicht stattfinden konnte; 17,0 % der Patienten gaben an, dass sich durch die SARS-CoV-2-Pandemie ihre Meinung gegenüber der Telemedizin geändert hat.

**Diskussion:**

Die Akzeptanz der Patienten für Telemedizin bei Patienten mit chronischem Offenwinkelglaukom scheint erstaunlich hoch. Diese ist durch die SARS-CoV-2-Pandemie noch weiter gesteigert worden. Diese Ergebnisse spiegeln eine generelle Bereitschaft bei Patienten mit chronischer Augenerkrankung wider, reflektieren jedoch nicht die Anwendbarkeit sowie die Akzeptanz aus ärztlicher Sicht. Diese Form der virtuellen Konsultation findet jedoch bei einem Großteil der Patienten mit Glaukom Akzeptanz und könnte überdacht werden.

## Abstract (en)

**Background:**

The COVID-19 pandemic in 2020 and 2021 severely restricted the care of ophthalmology patients. Teleophthalmological services, such as video consultation or medical telephone advice could, at least partially, compensate for the lack of necessary controls in the case of chronic diseases; however, teleophthalmological options are currently still significantly underrepresented in Germany.

**Objective:**

In order to determine the willingness of patients to use telemedicine and the virtual clinic, we conducted a survey using a questionnaire on the subject of teleophthalmology in university medicine patients with known glaucoma as a chronic disease during the first wave of the COVID-19 pandemic.

**Methods:**

A total of100 patients were interviewed. The questionnaire contained 22 questions with multiple choice possible answers. The inclusion criterion was the presence of glaucoma as a chronic disease, age over 18 years, and sufficient linguistic understanding to answer the questions. The data were collected, analyzed and anonymously evaluated.

**Results:**

In the patient survey it could be shown that the respondents with glaucoma are very willing to do teleophthalmology and that this would be utilized. Of the patients surveyed 74.0% would accept telemedicine and virtual clinics. Of the ophthalmological patients surveyed 54.0% stated that their visit to the doctor/clinic could not take place due to SARS-CoV‑2 and 17.0% of the patients stated that the SARS-CoV‑2 pandemic had changed their opinion of telemedicine.

**Discussion:**

The acceptance of telemedicine in patients with chronic open-angle glaucoma seems surprisingly high. This has been increased even further by the SARS-CoV‑2 pandemic. These results reflect a general willingness of patients with chronic eye disease but do not reflect the applicability and acceptance and applicability from a medical point of view; however, this form of virtual consultation is accepted by the majority of patients with glaucoma and could be considered for certain clinical pictures.

Die aktuelle Pandemie fordert das deutsche und die Gesundheitssysteme weltweit durch das neuartige SARS-CoV-2-Virus in vielerlei Weise heraus. Von den deutschen Gesundheitsbehörden wurden zeitgleich mit den Maßnahmen des Lockdowns Mitte März 2020 sowie erneut im November 2020 Kliniken angewiesen, alle elektiven Aufnahmen, Eingriffe und Operationen bis auf Weiteres zu verschieben, soweit dies medizinisch vertretbar war, um materielle und personelle Ressourcen für die Behandlung von COVID-19(Coronavirus SARS-CoV-2)-Patienten freizuhalten, aber auch um vermeidbare Übertragungswege des Virus zwischen Patienten und medizinischem Personal zu minimieren. Dies betraf wie alle anderen medizinischen Disziplinen auch ophthalmologisch Kliniken mit ihren stationären und ambulanten Leistungen. Auch im ambulanten Sektor entsteht so eine „weiche Triage“ bei der entschieden werden muss, welche Patienten gesehen werden und welche Patienten aufgeschoben werden können.

Zahlreiche Leistungen sind jedoch auch in Zeiten einer Pandemie dringlich aufrechtzuerhalten, s. auch WHO-Leitlinie „COVID-19: Operational guidance for maintaining essential health services during an outbreak“. Die Versorgung gefährdeter Bevölkerungsgruppen (Säuglinge und ältere Erwachsene), die Behandlung chronischer Krankheiten und das Management von Notfällen und akuten Patientenvorstellungen muss weiterhin gewährleistet sein [1]. Hierzu zählen auch ophthalmologische Erkrankungen. Zu den chronischen Erkrankungen zählt unter anderem das Glaukom bei dessen Therapie und Kontrollverzicht es zu chronischen oder auch akuten irreversiblen Schäden bis zur Erblindung kommen kann.

Eine Möglichkeit, diese essenzielle Gesundheitsdienstleistung und -versorgung der Ophthalmologie in gewissem Maße aufrechtzuerhalten und gleichzeitig zu einer Reduzierung des Infektionsrisikos durch SARS-CoV‑2 in Form des Social Distancing beizutragen, könnten telemedizinische Angebote sein. Unter Telemedizin (E-Health) wird die Verwendung von Telekommunikationstechnologien zum Austausch medizinischer Informationen für Diagnostik, Konsultation, Therapie und Lehre von Arzt zu Arzt und Arzt zu Patienten verstanden [2]. Bei den gesetzlichen Krankenkassen findet die Anwendung der Telemedizin zunehmend Zuspruch und ermöglicht im Zuge der elektronischen Krankenakte eine ganzheitliche Versorgung der Patienten. Hierdurch wird eine optimierte Vernetzung mit den behandelnden Augenärzten generiert und zusätzlich durch die digitale Übertragung von Patientendaten eine effektive, transparente Patientenversorgung gewährleistet.

Im angloamerikanischen Raum und auch Ländern wie Finnland mit geringer Bevölkerungsdichte finden telemedizinische, ophthalmologische Versorgungen bereits seit geraumer Zeit statt [3–6]. In Großbritannien wird die Telemedizin auch aufgrund des anderen Gesundheitssystems aktiv eingesetzt. Virtuelle Kliniken sind dort fester Bestandteil der Versorgung [3].

Auch in den USA findet die Telemedizin Anwendung im Bereich des Glaukoms oder Hornhautscreenings [6]. In Tokyo werden digitale Angebote zur Bestimmung von Indikationen für Hornhauttransplantationen ausgewertet [6].

Im Dezember 2019 wurde in Deutschland das vom Innovationsausschuss geförderte Projekt SALUS „Selbsttonometrie und Datentransfer bei Glaukompatienten zur Verbesserung der Versorgungssituation“ unter der Leitung von Frau Prof. Nicole Eter der Universitätsklinik Münster gestartet. Mit diesem Projekt soll die Versorgung von Glaukompatienten zukünftig verbessert werden, indem die Patienten den Augeninnendruck eigenständig in der häuslichen Umgebung messen, ohne hierfür stationär für die Erstellung eines Tagesdruckprofils in einer Klinik aufgenommen zu werden. Auch hier sind die Patienten, Kliniken und niedergelassenen Augenärzte dabei über eine elektronische Patientenakte telemedizinisch vernetzt [8]. Grundsätzlich bietet die Teleophthalmologie die Möglichkeit der schnellen und quervernetzten, z. B. videobasierten Unterstützung [4, 5].

Die Akzeptanz in der Bevölkerung zum Thema ophthalmologische Telemedizin ist im deutschsprachigen Raum bisher jedoch noch unklar, sodass wir hierzu eine Befragung betroffener Patienten am Beispiel einer chronischen Augenerkrankung des Glaukoms in die Wege geleitet haben, um die Akzeptanz gegenüber der Telemedizin objektiv evaluieren zu können. Hierfür wurden die Glaukompatienten mit einem standardisierten Fragebogen befragt, was sie von der telemedizinischen Versorgung erwarten und erhoffen, welche Bedenken sie haben. Laut unserer Recherche ist dies dabei die erste systematische Befragung chronisch kranker Glaukompatienten zum Aspekt Telemedizin in Deutschland.

## Methodik

**Figure 1:**
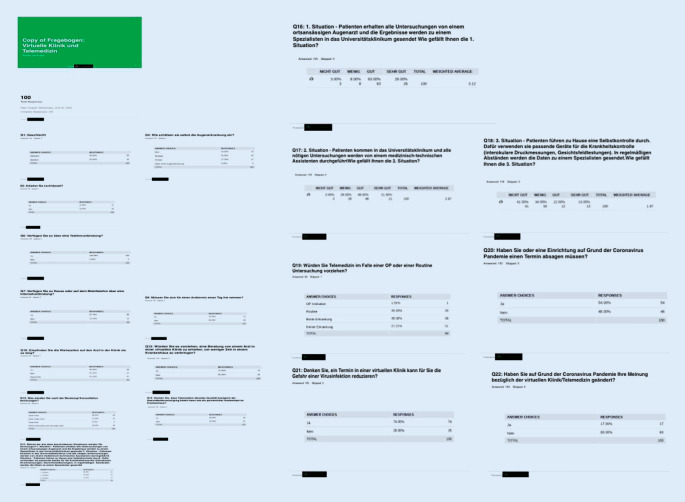

Es wurden 100 Patienten mit gesichertem Glaukom mittels eines Online Fragebogens über ein mobiles Tablet und der Software Survey Monkey® (Momentive Europe UC, Dublin, Irland) zum Thema ophthalmologische Telemedizin befragt. Alle Studienteilnehmer haben einen Fragenkatalog von insgesamt 22 Fragen erhalten. Die Fragen wurden untergliedert in allgemeine Fragen, Verfügbarkeit von Kommunikationsmedien, Stadium der chronischen Erkrankung, Akzeptanz im Allgemeinen, spezielle Präferenzen einer potenziellen virtuellen Klinik, Veränderungen durch die Pandemie (vgl. Abb. 1). Die Fragen konnten in Form von Einfach- und Mehrfachwahl mit ausreichend Bedenkzeit beantwortet werden. Die Daten wurden anonymisiert erhoben, analysiert und ausgewertet.

Als ein Einschlusskriterium galten das Vorhandensein eines Glaukoms, Alter über 18 Jahre sowie ein ausreichendes sprachliches und technisches Verständnis zur Beantwortung der Fragen.

## Ergebnisse

In der durchgeführten Studie wurden 100 Patienten mit bereits vor der SARS-CoV-2-Pandemie diagnostiziertem Glaukom zum Thema Telemedizin befragt. Hierbei haben gleichermaßen Frauen als auch Männer an der Studie teilgenommen (45,0 % vs. 55,0 %).

Von den befragten Personen befanden sich 47,0 % noch im Berufsleben, 53 % waren nicht berufstätig zum Zeitpunkt der Umfrage. Es wurde ermittelt, inwieweit die Patienten über eine Telefon- oder Internetverbindung verfügen, die sie für eine telemedizinische Beratung verwenden könnten. Hierbei zeigte sich eindeutig, dass 100 % der Patienten über eine Telefonverbindung und 87,76 % über eine Internetverbindung verfügen, im privaten Umfeld daheim oder mobil auf dem Smartphone; 12,2 % der Befragten gaben an, keinen validen Internetzugang zu besitzen.

**Figure 2:**
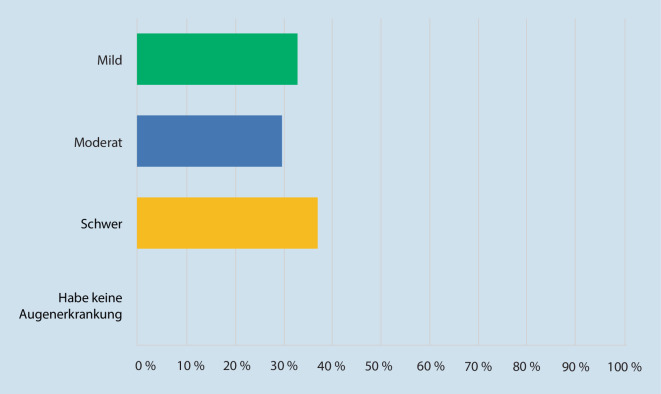

Bezüglich des Schweregrads der chronischen Erkrankung Glaukom gaben 33,0 % der Befragten subjektiv an, dass sie an einer milden Ausprägung des Glaukoms leiden; 30,0 % der Befragten stuften ihre Augenerkrankung als moderat ein; 37,0 % der Befragten empfanden, dass sie schwer an Glaukom erkrankt waren (vgl. Abb. 2).

**Figure 3:**
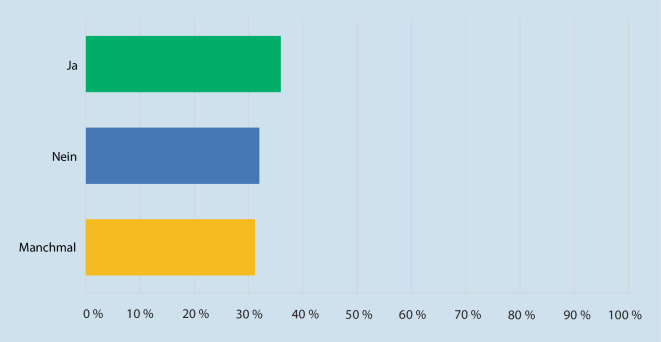

Um einen Arzttermin wahrnehmen zu können, müssen 32,0 % der Befragten einen Urlaubstag einplanen, da es häufig zu verlängerten Wartezeiten beim Augenarzt kommt. Hierbei empfinden 36,3 % der Patienten die Wartezeit stets als zu lang, 31,3 % geben an, dass die Wartezeit manchmal zu lang ist; 70,0 % der Befragten würden einer virtuellen Klinik den Vorzug geben, um die Wartezeit zu verkürzen und den Aufenthalt in einer Klinik zur ambulanten Kontrolle zu reduzieren (vgl. Abb. 3 und 4).

**Figure 4:**
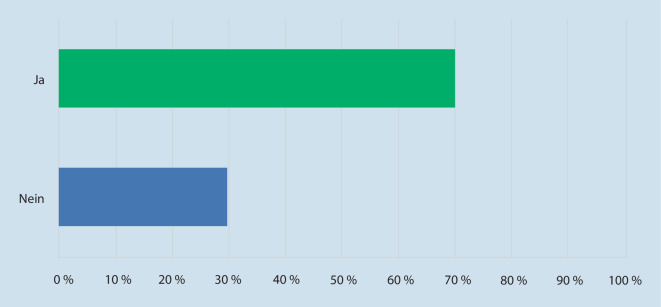

Hierbei sprechen sich 36,0 % der Patienten für eine telefonische ärztliche Konsultation aus, 31,0 % präferieren eine ärztliche Beratung über Videoanruf. In 28,0 % der Fälle würden die Patienten gerne zusätzlich zu einem Telefonat oder einer Videokonferenz einen Arztbrief mit den Befunden und der Therapie erhalten. Lediglich 5 % der befragten Personen geben an, dass ein regelmäßiger Briefkontakt mit dem Arzt ausreichen würde. Allerdings gaben 62,0 % der Patienten an, dass die Telemedizin nicht dieselbe Qualität bezüglich der Gesundheitsversorgung bieten kann wie ein persönlicher Kontakt zum Arzt trotz der hohen Bereitschaft zur Telemedizin.

Es würden 63,0 % der Befragten eine Form der virtuellen Klinik befürworten, bei der alle Untersuchungen von einem ortsansässigen Augenarzt gemacht würden und im Folgenden die Ergebnisse der Untersuchungen zu einem Spezialisten in dem Universitätsklinikum gesendet würden und begutachtet würden; 26,0 % der Patienten fanden dies eine sehr gute Idee, die sie gerne wahrnehmen würden, wenn dieses Angebot bestehen würde.

Als Nächstes wurde den Umfrageteilnehmern vorgeschlagen, dass die Patienten zu Hause eigenständig einen Selbsttest durchführen mit passenden Geräten für die Krankheitskontrolle (beispielsweise in diesem Fall Druckmessungen und Gesichtsfeldtestungen). Diese Messungen würden in regelmäßigen Abständen zu einem ophthalmologischen Spezialisten gesendet und bewertet werden. Anhand dieser Daten würde eine medizinische Versorgung des Patienten in die Wege geleitet. Hierfür sprachen sich 35,0 % der befragten Patienten aus (vgl. Abb. 4). Gleichzeitig gaben 39,9 % der Patienten an, dass sie Telemedizin gegenüber einer Routineuntersuchung vorziehen würden. Im Rahmen der SARS-CoV-2-Virus-Pandemie gaben 54,0 % der Befragten an, dass sie ihren Arzt‑/Klinikbesuch absagen mussten oder dieser abgesagt wurde. Hierbei geben 74,0 % der Befragten an, dass sie hier einen deutlichen Vorteil in der Telemedizin sehen würden. Die Gefahr einer Virusverbreitung und Ansteckung mit einer Virusinfektion sei so reduziert; 17,0 % der Patienten gaben an, dass sich durch die Pandemie ihre Meinung gegenüber der Telemedizin geändert hat.

## Diskussion

Während der COVID-19-Pandemie mussten Patienten, aber auch klinische Einrichtungen multiple Einschränkungen hinnehmen. Diese erschwerten die Untersuchung und die Behandlung der Patienten. Planbare Konsultationen und Eingriffe sollten möglichst aufgeschoben werden, um Ressourcen für Intensivstationen freizuhalten und Kontakte zu reduzieren. Dadurch entstand und entsteht eine „weiche Triage“. Trotzdem muss eine Grundversorgung sowohl bei akuten als auch bei chronischen Erkrankungen wie dem Glaukom, aber auch bei anderen ophthalmologischen Erkrankungen auch in Krisensituationen gewährleistet sein.

Eine Möglichkeit, diese essenziellen Gesundheitsdienstleistungen und -versorgung der Ophthalmologie aufrechtzuerhalten und zu einer Reduzierung des Infektionsrisikos beizutragen, könnten telemedizinische Angebote sein. Bei den gesetzlichen Krankenkassen findet Telemedizin zunehmend Zuspruch und ermöglicht eine ganzheitliche Versorgung der Patienten, eine optimierte Vernetzung der behandelnden Ärzte und gewährleistet eine effektive, transparente Patientenversorgung.

Im angloamerikanischen Raum und auch Ländern wie Finnland mit geringer Bevölkerungsdichte finden telemedizinische, ophthalmologische Versorgungen bereits seit geraumer Zeit statt [3–6]. In Großbritannien wird die Telemedizin auch aufgrund des anderen Gesundheitssystems aktiv eingesetzt: Der Opticians Act 1989 bestimmte britische Optometristen zu Routinekontrollen für Augenveränderungen. Im Falle von Auffälligkeiten werden Ärzte virtuell konsultiert. Ziel war es, das britische Gesundheitssystem, den National Health Service (NHS), personell und ökonomisch zu entlasten. Virtuelle Kliniken sind dort fester Bestandteil der Versorgung [3].

Ein anderes Beispiel liefert die University of Texas. Hier findet z. B. die Telemedizin Anwendung im Bereich des Glaukomscreenings, bei dem digitale Bilder des Papillenkopfes aufgenommen werden. Gleichzeitig werden die Augeninnendruckniveaus bei diesem Projekt mithilfe digitaler Messgeräte gemessen und alle erhobenen Befunde digital durch Augenärzte aus Fachkliniken überprüft [6]. In der Abteilung für Augenheilkunde des Tokyo Dental College werden so auch digitale Spaltlampenbilder zur Bestimmung von Indikationen für Hornhauttransplantation ausgewertet sowie hierdurch seltene korneale Augenerkrankungen oder Komplikationen von Hornhauterkrankungen dokumentiert. Die Telemedizin findet im Bereich des Vorderabschnitts noch viele weitere Anwendungsmöglichkeiten, so z. B. auch im Bereich des Astigmatismusscreenings an der University of Arizona [6].

Grundsätzlich bietet die Teleophthalmologie die Möglichkeit der schnellen und quervernetzten, z. B. videobasierten Unterstützung [4, 5]. Die Anwendung in Deutschland und in der Ophthalmologie hinkt jedoch noch hinterher. Dabei stellt sich neben den Schwierigkeiten der Patientenselektion und den Fehlerquellen des virtuellen Settings gerade auch bei Follow-up-Patienten für eine virtuelle Klinik natürlich auch die Frage der Akzeptanz der Patienten generell. Die richtige Auswahl der Patienten beinhaltet sicherlich viele Fallstricke.

Ziel unserer Arbeit war es, die Akzeptanz von Glaukompatienten für eine solche virtuelle Klinik zu erfragen. Klar nicht erfasst in dieser Studie war die Durchführbarkeit einer solchen virtuellen Diagnostik und Behandlung der Glaukompatienten. Dies würde, wie oben beschrieben, sicherlich andere zu berücksichtigende Fallstricke liefern. Interessanterweise lieferte unsere Umfrage folgende Ergebnisse: Die kommunikative Infrastruktur ist bei den Patienten gegeben (Telefon, Internet); 75 % der Patienten stehen einer virtuellen Klinik positiv gegenüber; Patienten bevorzugen Telefonanrufe; ein virtuelles Setting mit Untersuchungen beim Hausarzt statt Selbsttest werden bevorzugt; die Pandemie hat die Akzeptanz noch weiter verstärkt.

Es zeigten 75 % unserer Befragten eine grundlegende hohe Akzeptanz gegenüber der Telemedizin. Die Pandemie hat zur weiteren Akzeptanz in ca. 20 % beigetragen. Verlängerte Wartezeiten und Urlaubsnahme spielen hierbei, wie im Fragebogen erhoben, eine zusätzliche Rolle. Über 50 % der Patienten waren noch berufstätig. Limitierend muss hier festgestellt werden, dass es sich bei dem Setting um Befragte in der Universitätsmedizin als Tertiärversorger handelte, bei denen die Anreise- und Wartezeit natürlich deutlich länger sind als bei dem lokalen niedergelassenen Augenarzt. Bei der Befragung, welches virtuelle Setting bevorzugt würde (Selbstscreening zu Hause oder Abwickeln der Befunde über den eigenen Augenarzt statt Überweisung in die Klinik), präferierten nur 30 % die Selbsttestung im Allgemeinen, während über 60 % eine reine Betreuung durch den Niedergelassenen bevorzugen würden. Hier liegt sicherlich ein deutlicher Bias vor, und eine Umfrage im Niedergelassenensetting müsste erfolgen. Hier zeigt sich ggf. ein deutlich anderes Bild. Unsere Befragung hat auch ergeben, dass die Infrastruktur (Telefon und Internet) grundsätzlich zu 100 % vorhanden ist. Limitierend ist auch hier jedoch zu sagen, dass nur Internet generell abgefragt wurde, ohne die Geschwindigkeit und das Ausreichen für eine gute Verbindung zu erfragen. Hier wäre ggf. ein weiterer Ausbau notwendig. Interessanterweise bevorzugten jedoch mehr Patienten laut unserer Umfrage die telefonische Beratung. Die Gründe können jedoch vielfältig sein wie Privatsphäre, Konzentration oder auch Technik. Auf der anderen Seite ermöglichen Videosprechstunden jedoch in hoher Auflösung zusätzlich v. a. Untersuchungen. So können neben den externen Adnexen, Pupillenreaktion, Motilität, Ausrichtung der Lichtreflexe des vorderen Augenabschnitts, der Iris und der Hornhaut (spezielle externe Lichtquelle notwendig) vom Patienten und unter Anweisung des Arztes untersucht verwendet [7, 8]. Um eine personalisierte Telemedizin anbieten zu können, sollten den Patienten sowohl Telefonanrufe als auch Videovisiten angeboten werden. Videovisiten sind aufgrund des Videos für die Triage von Patienten auf jeden Fall von Vorteil. Es muss jedoch bedacht werden, dass eine patientengenerierte Bildinterpretation für den behandelnden Arzt entsteht. Bei der Erkrankung des Glaukoms sind Videokonsultationen besonders nützlich für den Medikamentenabgleich und z. B. zur Beurteilung der Glaukommedikamententoleranz [9]. Um die Akzeptanz für die Telemedizin nicht nur seitens der Patienten, sondern auch für die jeweiligen Anbieter zu erhöhen, ist es erforderlich, dass die erbrachten telemedizinischen Leistungen auch leistungsorientiert vergütet werden. Aktuell können z. B. ärztlich beratende Telefonate mit Patienten, anders als in anderen Ländern, oft nicht adäquat in Rechnung gestellt werden [10, 11].

Wie oben bereits erwähnt finden die reine Selbsttestung und anschließende Remote-Konsultation jedoch nur bei 30 % der Patienten Akzeptanz. Solche Anwendungsmöglichkeiten gibt es bereits. Bei Testverfahren zur Sehschärfenbestimmung können im Rahmen der telemedizinischen Versorgung mobile Apps als Selbsttest Anwendung finden und die so erhobenen Daten mithilfe von Verlaufsdiagrammen verifiziert werden. Diese Messwerte können dann in der Fernauswertung ärztlich validiert werden. Jedoch muss die Durchsetzbarkeit der häuslichen Tonometriemessung gewährleistet sein und eine Reliabilität von IOD-Messungen gesichert und bewahrt werden. Zusätzlich können ebenso mobile Amsler-Raster bei Erkrankungen der Netzhaut Anwendung finden und digital im Verlauf dokumentiert und online für den Ophthalmologen verfügbar gemacht werden. Hierfür gibt es bereits erste mobile und Online-Refraktionstools, die dieses ermöglichen. Im Dezember 2019 wurde in Deutschland das vom Innovationsausschuss geförderte Projekt SALUS „Selbsttonometrie und Datentransfer bei Glaukompatienten zur Verbesserung der Versorgungssituation“ unter der Leitung von Frau Prof. Nicole Eter der Universitätsklinik Münster gestartet. Mit diesem Projekt soll die Versorgung von Glaukompatienten zukünftig verbessert werden, indem die Patienten den Augeninnendruck eigenständig in der häuslichen Umgebung messen, ohne hierfür stationär für die Erstellung eines Tagesdruckprofils in einer Klinik aufgenommen zu werden. Auch hier sind die Patienten, Kliniken und niedergelassenen Augenärzte dabei über eine elektronische Patientenakte telemedizinisch vernetzt [12].

Zusammenfassend ist eine große Bereitschaft für Telemedizin bei chronischen Glaukompatienten grundsätzlich vorhanden. Limitierend muss man jedoch sagen, dass diese Umfrage Befragte in einem Tertiärversorgungszentrum darstellt. Die Primärversorgungsumfrage könnte ganz anders aussehen. Eine generelle Akzeptanz der Patienten ist jedoch da, und eine solche Versorgung kann sicherlich Versorgungsengpässe in gewissem Maße abdecken. Die Patientenakzeptanz spiegelt jedoch nicht die Fallstricke der richtigen Selektion seitens der Ärzte wider, um auch den richtigen Patienten für die entsprechende Versorgung zu wählen. Hier könnte sich ein ganz anderes Bild zeichnen. Eine weitere Schwachstelle dieser Befragung ist die Patientenselektion. Es handelte sich in diesem Fall lediglich um Glaukompatienten, die eine chronische Erkrankung haben. Andere ophthalmologische – gerade auch akute Krankheitsbilder – wurden nicht erfasst. Wie oben bereits beschrieben, handelt es sich hierbei lediglich um einen Fragebogen zur Akzeptanz. Eine Umsetzbarkeit in der Klinik mit guter Umsetzbarkeit im virtuellen Setting bedeutet dies nicht. Hierbei sind ganz andere Hürden zu überwinden, wie z. B. die Sicherstellung der Follow-ups dieser Patienten mit guter struktureller und funktioneller Progressionsanalyse. Des Weiteren muss sichergestellt werden, dass auch der Augeninnendruck reliabel zu Hause gemessen werden kann. Hier fehlt es noch an guten, reliablen Geräten. Für den Patienten klingt es demnach zunächst einfacher, sich vor den Computer zu setzen und die Untersuchungen remote durchzuführen und dann zur passenden Zeit angerufen zu werden. Hier müssen jedoch auch der Benefit und die Reliabilität dieses Follow-ups sichergestellt werden. Die sichere Umsetzung einer virtuellen Klinik verlangt daher noch eine Menge Arbeit aus ophthalmologischer Sicht. Dabei muss man aber sicherlich auch die Art der Umsetzung der virtuellen Klinik unterscheiden. Eine reine Remote-virtuelle Klinik seitens des Patienten ist sicherlich weiter davon entfernt, umsetzbar zu werden, als ein Remote-Austausch der Befunde zwischen niedergelassenem Augenarzt und tertiärem Versorgungszentrum.

## Fazit für die Praxis

Die virtuelle Klinik stellt ein innovatives Konzept zur Patientenversorgung bei chronischen ophthalmologischen Erkrankungen und Verlaufskontrollen dar. Es besteht eine zufolge unserer Umfrage hohe Bereitschaft zur Teleophthalmologie, die von den Patienten gerne in Anspruch genommen werden würde.
